# Identification of Genomic Regions and Candidate Genes for Litter Traits in French Large White Pigs Using Genome-Wide Association Studies

**DOI:** 10.3390/ani12121584

**Published:** 2022-06-19

**Authors:** Jianmei Chen, Ziyi Wu, Ruxue Chen, Zhihui Huang, Xuelei Han, Ruimin Qiao, Kejun Wang, Feng Yang, Xin-Jian Li, Xiu-Ling Li

**Affiliations:** College of Animal Science and Technology, Henan Agricultural University, Zhengzhou 450046, China; jmchen48119@126.com (J.C.); w17746978907@163.com (Z.W.); chenruxue2022@163.com (R.C.); huangangg@163.com (Z.H.); hxl014@126.com (X.H.); qrm480@163.com (R.Q.); wangkejun.me@163.com (K.W.); chinafy6868@163.com (F.Y.); lxjlongfei@163.com (X.-J.L.)

**Keywords:** pig, reproductive traits, SsGWAS, SNP, genetic variants

## Abstract

**Simple Summary:**

Sow reproductive performance is crucial in the entire pig industry. However, the heritability of reproductive traits is slowthat it is difficult to make great genetic progress by traditional breeding methods. Therefore, in this study, we performed genome-wide association analyses of litter traits in French White sows to explore genes related to sow reproductive traits and we finally detected a notable region (10.72–11.06 Mb on chromosome 7) and four promising genes (*JARID2*, *PDIA6*, *FLRT2* and *DICER1*). The results of this study provide a theoretical basis for subsequent molecular breeding.

**Abstract:**

The reproductive traits of sows are one of the important economic traits in pig production, and their performance directly affects the economic benefits of the entire pig industry. In this study, a total of 895 French Large White pigs were genotyped by GeneSeek Porcine 50K SNP Beadchip and four phenotypic traits of 1407 pigs were recorded, including total number born (TNB), number born alive (NBA), number healthy piglets (NHP) and litter weight born alive (LWB). To identify genomic regions and genes for these traits, we used two approaches: a single-locus genome-wide association study (GWAS) and a single-step GWAS (ssGWAS). Overall, a total of five SNPs and 36 genomic regions were identified by single-locus GWAS and ssGWAS, respectively. Notably, fourof all five significant SNPs were located in 10.72–11.06 Mb on chromosome 7, were also identified by ssGWAS. These regions explained the highest or second highest genetic variance in the TNB, NBA and NHP traits and harbor the protein coding gene *ENSSSCG00000042180*. In addition, several candidate genes associated with litter traits were identified, including *JARID2*, *PDIA6*, *FLRT2* and *DICER1*. Overall, these novel results reflect the polygenic genetic architecture of the litter traits and provide a theoretical reference for the following implementation of molecular breeding.

## 1. Introduction

The reproductive capacity of sows plays an important role in the composition of the economic benefits of the pig industry. In order to obtain more profit, it is especially important to improve the sow reproductive performance. Litter traits, such as total number born (TNB), the number born alive (NBA), the number of healthy piglets (NHP) [1] and the litter weight birth alive (LWB) are critical indexes to evaluate the reproductive performance of sows. However, the genetic architecture of litter traits is much more complicated because of low heritability (~0.1) [2]. In the studies of Large White pigs, Canario et. al. reported that the heritabilities of TNB, and NBA were 0.10 and 0.08, respectively [3]. The heritabilities of TNB, NBA, NHP, and LWB were 0.07, 0.06, 0.05 and 0.06, respectively, in the study that was reported by Ye et al. [1]. Therefore, the genetic improvement obtained by traditional breeding programs is very slow. Subsequently, the marker-assisted selection (MAS) and genomic selection (GS) are highly encouraged and have improved the genetic gain of reproduction traits [4].

During the recent decades, with the development of molecular breeding technology, genome-wide association study (GWAS) has been widely used to identify quantitative trait loci (QTL) and quantitative trait nucleotides (QTN) in complex traits. In pigs, GWAS has become one of the most powerful methods for exploring single nucleotide polymorphisms (SNPs) and candidate genes associated with complex traits [5,6,7,8]. In recent decades, many reproductive related genes have been identified by the GWAS method. Most of them are hormones secreted by the gonads and their receptors, embryonic development related genes, such as including *ESR*, *FSHβ*, *PRLR* and *RFRP* [9,10,11,12]. Although the traditional single-locus GWAS could identify several SNPs that were statistically significant. But there wasstill a proportion of SNPs with small effects that failed to reach significant levels and waslost. Among the statistical methods for GWAS, single-step GWAS (ssGWAS) has been performed for pig economical traits [5,13,14]. For this approach, all genotypes, all phenotypes and all pedigree information are simultaneously incorporated into a mixed linear model and calculated in a single step. Additionally, ssGWAS used a window composed of consecutive SNPs can efficiently improve the call rate of SNPs with small effect values, and it also can reduce noise and highlight the most significant peaks by refining iterative SNP weights [15]. We use two methods to verify and supplement mutually and expect more convincing results.

However, reproduction traits are controlled by many minor genes, there are only few candidate genes that have been identified until now and just explain a small proportion of genetic variance. Therefore, it is necessary to further investigate the genetic mechanism for reproductive traits. In this study, to identify candidate regions/genes and explore genetic architecture of litter traits (TNB, NBA, NHP and LWB), the traditional GWAS and ssGWAS methods were performed on 895 French Large White pigs genotyped by GeneSeek Genomic Profiler (GGP) 50K Porcine Beadchip.

## 2. Material and Methods

### 2.1. Animal and Phenotypic Data

In this study, a total of 1407 French Large White pigs were collected from a core pig breeding farm and were grouped in standard commercial pens. Each pig was bred under the same forage and feeding management conditions. All phenotypic records came from the first parity. The traits included TNB, NBA, NHP and LWB. NHP is the number of piglets with a birth weight above 1 kg. Four traits were recorded from January 2018 to March 2022. All 1407 sows have pedigree information. The NBA <3 and TNB >25 were excluded [16] and the normal distribution was checked before analysis. The averages, standard deviations, minimum values and maximum values of phenotypic records for TNB, NBA, NHP and LWB are listed in Table 1.

### 2.2. Genomic Data and Quality Control

Genomic DNA of 895 sows was extracted from the ear tissue using a genome extraction kit (Wuhan NanoMagBio Technology Co., Ltd., Wuhan, China) and genotyped using the GGP 50K SNP Beadchip (Neogen Corporatuib, Lansing, MI, USA). Data quality control was performed by PLINK v1.90 [17]. SNP markers with genotype call rate <0.90, minor allele frequency <0.05 and *p* < 10^−6^ for Hardy–Weinberg equilibrium test were filtered out. Individuals were filtered out with SNP marker call rates <0.90. All SNPs located in unmapped regions were also excluded. Subsequently, a final set of 895 individuals and 42726 SNPs was retained for subsequent analyses.

### 2.3. Estimation of Genetic Parameters

The AIREMLF90 module of BLUPF90 software family was used to estimate the genetic parameters of all traits [18]. The variance components were estimated using the restricted maximum likelihood (REML) approach. A univariate REML analysis led to the assessment of the heritability and a bivariate REML analysis was used to calculate genetic and phenotypic correlations between traits. The statistical model was:

y=Xβ+Zu+e

where y is the phenotypic vector; β is the vector of fixed effects. The fixed effect used in this study was the year-season; u is a vector of additive genetic effects and $u\;\sim \;N\;\left(0,H\sigma_{a}^{2}\right)$, in which H is the hybrid relationship matrix that combines pedigree and genomic relationships; e is a vector of residual effects and $e\;\sim \;N\;\left(0,I\sigma_{e}^{2}\right)$. X and Z are incidence matrices for β and u respectively. The inverse of H $\left(H^{-1}\right)$ was computed as [19]:

$$H^{-1}=A^{-1}+\left[\begin{array}{cc} 0 & 0\\ 0 & G^{-1}-A_{22}^{-1} \end{array}\right]$$

in which: A is the numerator relationship matrix based on pedigree for all animals, $A_{22}$ is the subset of the numerator relationship matrix for genotyped individuals and G is the genomic relationship matrix. The superscript −1 indicates that it is the inverse of the correlation matrix.

### 2.4. Genome-Wide Association Study

The GWAS was performed by the R package rMVP using the mixed linear model [20]. The statistical model was:

y=Wb+Za+Sc+e

in which y is the vector of phenotypic observations; b is the vector of fixed effects which includes year-season; a is the vector of the SNP substitution effects; c is the vector of additive genetic effects and $c\;\sim \;N\;\left(0,G\sigma_{a}^{2}\right)$, where G is the genomic relationship matrix, $\sigma_{a}^{2}$ is additive variance; e is a vector of residual effects and $e\;\sim \;N\;\left(0,I\sigma_{e}^{2}\right)$. W, Z and S are the incidence matrices for b, a and c, respectively. A genome-wide suggestive significant threshold value based on the Bonferroni method was defined as 1/N, where N is the number of SNPs after quality control.

### 2.5. Single-Step Genome-Wide Association Study

The ssGWAS was performed using BLUPF90 software family [19]. The RENUMF90 module was used to prepare phenotype and pedigree data, then we ran BLUPF90 module to calculate Genomic Estimated Breeding Values (GEBVs). Finally, the POSTGSF90 module was used to convert from GEBVs to SNPs effects and to calculate the variance explained by adjacent windows. The statistical model used in ssGWAS was the same as the model used in estimation of genetic parameters. The equation for converting GEBVs to SNPs effects was [15]:

$$\hat{u}=qDZ^{\prime }{\left[ZDZ^{\prime }q\right]}^{-1}{\hat{a}}_{g}$$

where Z is an incidence matrix of gene content adjusted for allele frequencies, D is a diagonal matrix of weights for variances of SNP effects (initially D=I), q is the weighting factor, ${\hat{a}}_{g}$ is the GEBVs of genotyped animals.

The process ofcalculating SNPs effects was iterated twice. The percentage of genetic variance explained by i-th consecutive SNPs (SNP window) was calculated as described by Wang et al. [15]:

$$\frac{Var\left(\sum_{j=1}^{5}Z_{j}{\hat{u}}_{j}\right)}{\sigma_{a}^{2}}\times 100\%$$

where $\sigma_{a}^{2}$ is the total genetic additive variance, $Z_{j}$ is a vector of the gene content of the *j*-th SNP for all animals and ${\hat{u}}_{j}$ is marker effect of the *j*-th SNP.

Beissinger et al. [21] reported that on matter sliding windows consisted of 5 or 10 SNPs, the ratio of detection rate to false-positive rate is most favorable over larger window sizes. In this study, we computed the proportions of the additive genetic variance that was explained by sliding windows of 5 adjacent SNPs.

### 2.6. Annotation of Genomic Regions and Search for Genes

For GWAS, candidate genes were defined as the genes that were nearest the significant SNPs. In the ssGWAS, genomic windows that explained >1% were considered to be relevant for all traits [22]. All types of genes (protein coding, snoRNA, lncRNA, miRNA, snRNA, Y_RNA, pseudogene) within the candidate windows or partly overlapped were searched on the Sscrofa11.1 genome (http://www.ensembl.org/, accessed on 24 April 2022), and only the protein coding genes were used for the following analysis. Linkage disequilibrium (LD) analysis of overlapping regions identified by two methods was performed by Haploview 4.2 software [23]. We manually searched literature to identify genes associated with reproductive traits as candidate genes. Previously identified QTL in the pig genome was evaluated using the PigQTLdb release 46 in 27 December 2021 [24].

## 3. Results

### 3.1. Phenotypic Statistics

The descriptive statistics of four reproductive traits are listed in Table 1. The TNB, NBA and NHP range from 1 to 25 and the mean values are 14.22, 12.77 and 12, respectively. The minimum, maximum and mean values of LWB are 2.6, 27.9 and 15.31 kg, respectively. Moreover, the coefficient of variation (CV) values were higher for all traits, which shows that the data are discrete to some extent.

### 3.2. Genetic Parameter Statistics

The heritability, genetic and phenotypic correlations of the four litter traits were estimated (Table 2). In general, the heritability for TNB, NBA and LWB ranged from 0.06 to 0.13. The heritability of NHP was 0.02. Genetic correlations of all traits were highly positively correlated, ranging from 0.70 to 0.99, except for a lower genetic correlation of 0.38 between TNB and LWB. Additionally, the four traits were highly positively correlated with the phenotypic correlations ranged from 0.68 to 0.93.

### 3.3. Traditional GWAS for Litter Traits

**Figure 1:**
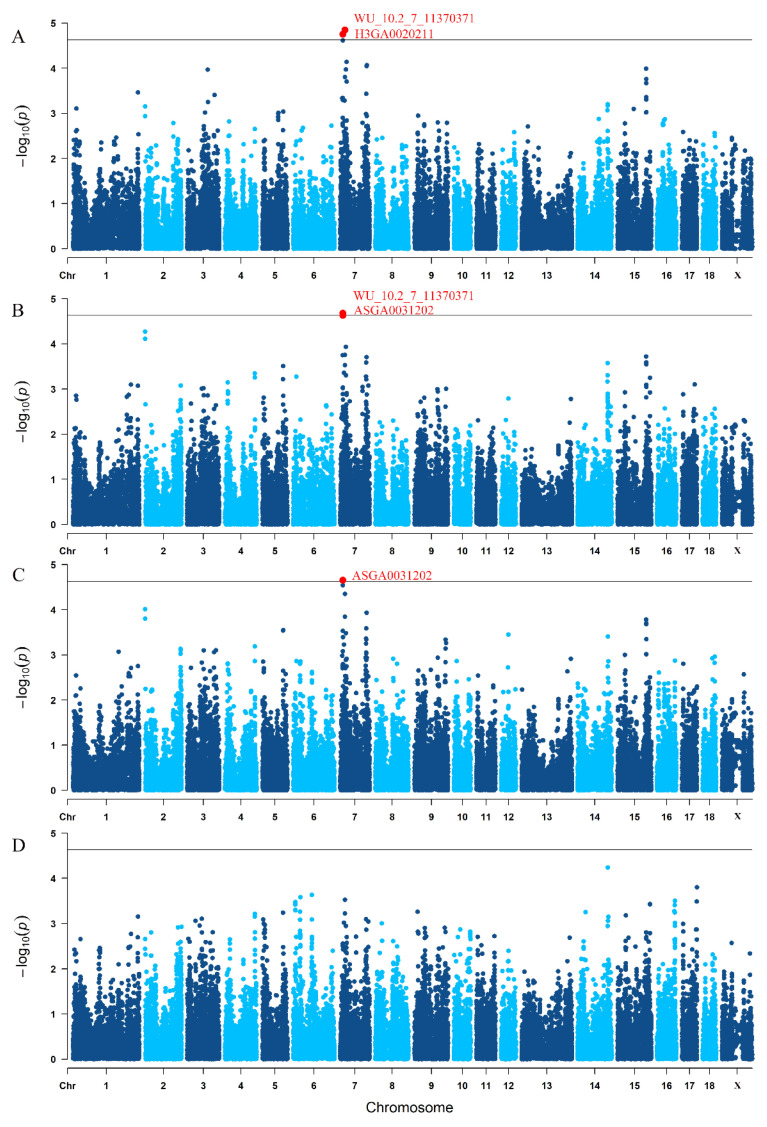
The Manhattan plots of genome-wide association study for four reproductive traits. (**A**) TNB: total number born; (**B**) NBA: number born alive; (**C**) NHP, number healthy piglets; (**D**) LWB: litter weight born alive. Each dot represents one SNP. The y-axis represents −log10(P). The x-axis represents the position of SNPs. The horizontal solid line depicts the chromosome-level significance threshold value.

There were 2, 2, 1 and 0 SNPs that reached a chromosome-level significance for TNB, NBA, NHP and LWB, respectively (Figure 1). These significant SNPs are all located on Sus scrofa chromosome (SSC) 7. Among five significant SNPs, two SNPs (WU_10.2_7_11370371 and ASGA0031202) associated with TNB, NBA and NHB were repeated, and they were both located around 10.8 Mb on SSC 7. Furthermore, another SNP (H3GA0020211) associated with TNB was located in 19.6 Mb on SSC 7 (Table 3).

### 3.4. Single-Step GWAS for Litter Traits

**Figure 2:**
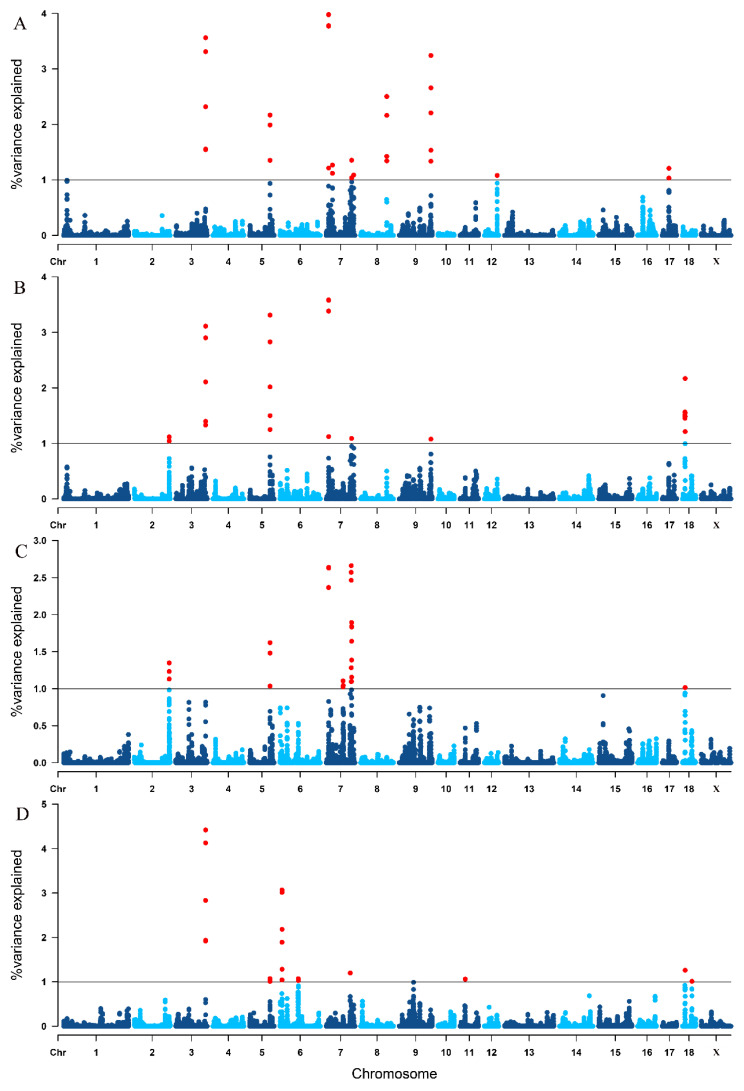
The Manhattan plots of single-step genome-wide association study for four reproductive traits. (**A**) TNB: total number born; (**B**) NBA: number born alive; (**C**) NHP, number healthy piglets; (**D**) LWB: litter weight born alive. Each dot represents one window of 10 adjacent SNPs. The y-axis displays the proportion of genetic variances of each window. The x-axis represents the position of windows for chromosomes. The horizontal solid line depicts the significance level (proportion of genetic variances = 1%).

There were 42,726 windows for ssGWAS analysis after dividing five consecutive SNPs into one window, of which, the last nine windows contained fewer than five SNPs. We identified 32, 26, 28 and 22 relevant 5-SNP windows which explained 1% or more of the total genetic variance for TNB, NBA, NHP and LWB, respectively (Figure 2 and Table S1). Table 4 shows the significant genomic regions for four litter traits. For TNB, 10 genomic regions which distributed on SSC 3, 5, 7, 8, 9, 12 and 17, and the highest region of 10.72–11.06 Mb on SSC 7 explained 3.979% of genetic variance. There were eight genomic regions that were significantly associated with NBA and they distributed on SSC 2, 3, 5, 7, 9 and 18. The highest region was also located in 10.72–11.06 Mb on SSC7 and it accounted for 3.585% of genetic variance. For NHP, 28 windows concatenated into 10 genomic regions. These genomic regions distributed on SSC 2, 5, 7 and 18. The second highest region explained 2.640% of genetic variance and it was similarly located at 10.72–11.03 Mb on SSC7. We obtained eight genomic regions associated with LWB and they distributed on SSC 3, 5, 6, 7, 11 and 18 (Table 4).

### 3.5. Identification of Candidate Genes

**Figure 3:**
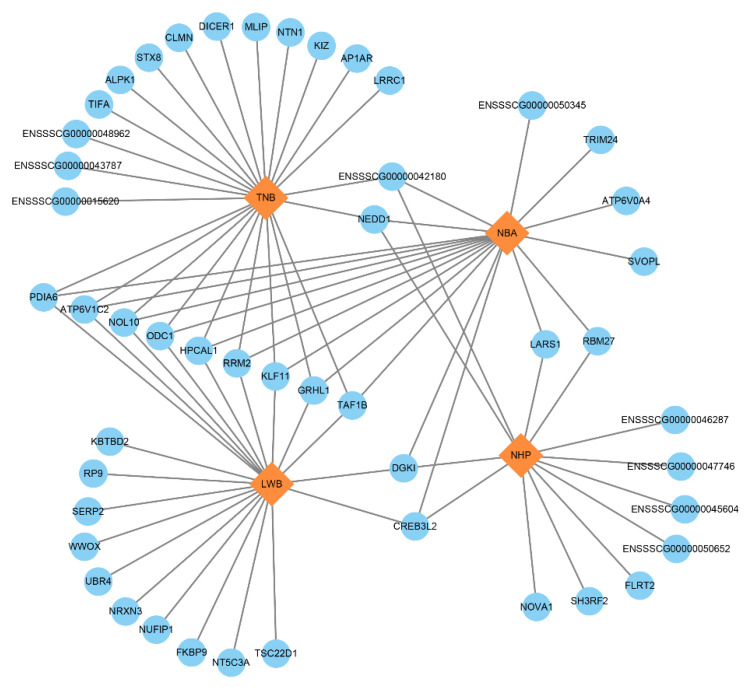
The 49 protein coding genes related to four reproductive traits. The blue circles represent genes and the orange diamonds represent traits. TNB: total number born; NBA: number born alive; NHP: number healthy piglets; LWB: litter weight born alive.

**Figure 4:**
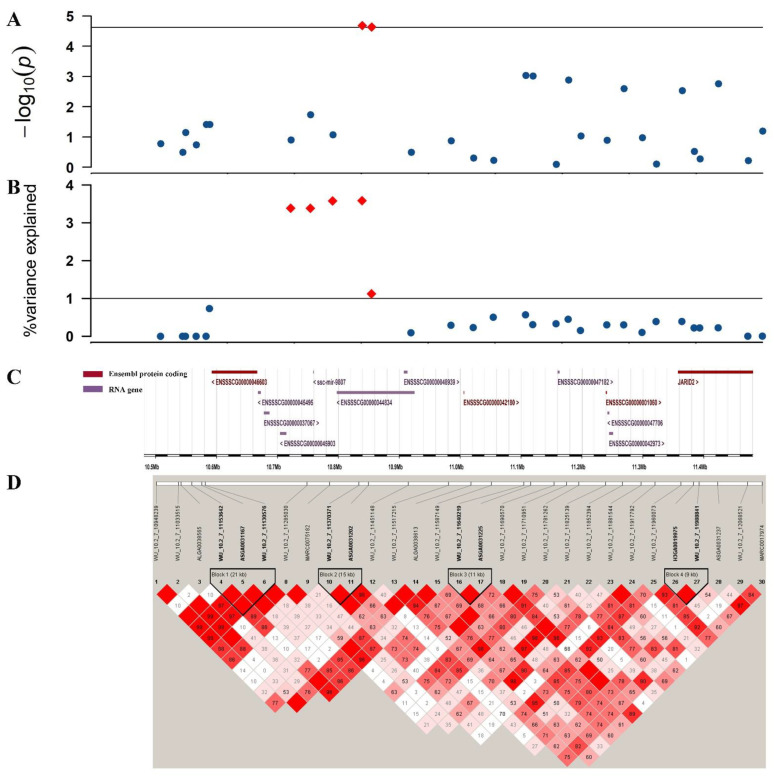
The analysis of 10.5–11.5 Mb region. (**A**) The Manhattan plot of genome-wide association study for NBA trait; (**B**) The Manhattan plot of single-step genome-wide association study for NBA trait; (**C**) genes distributed in 10.5–11.5 Mb region; (**D**) linkage disequilibrium analysis for 10.5–11.5 Mb region.

The results of traditional GWAS in TNB, NBA and NHP traits discovered two adjacent SNPs (WU_10.2_7_11370371 and ASGA0031202) located on 10.8 Mb of SSC7. We annotated the *JARID2* gene that was located 0.5 Mb downstream of these two SNPs. Another candidate gene was *GMNN,* which was located 0.07 Mb upstream of the SNP (H3GA0020211). In ssGWAS, we obtained 82 genes totallyfor all four reproductive traits, including 49 protein coding genes (Figure 3 and Table S2). In which, nine genes (*PDIA6*, *ATP6V1C2*, *NOL10*, *ODC1*, *HPCAL1*, *RRM2*, *KLF11*, *GRHL1* and *TAF1B*) were related to the TNB, NBA and LWB traits and the *DGK1* and *CREB3L2* genes were associated with the NBA, NHP and LWB traits. Two genes (*ENSSSCG00000042180* and *NEDD1*) were simultaneously associated with the TNB, NBA and NHP traits. For the location of 10.8 Mb, which was detected in both methods, we selected the 10.5–11.5 Mb region of the NBA trait in its vicinity for analysis (Figure 4). We annotated four protein coding genes and there were four LD blocks in this region. For all genes, we manually searched the literature to discover if they had a previously identified relationship with the reproductive traits under study and the results of candidate genes are listed in Table 5.

## 4. Discussion

Litter traits contained the TNB, NBA, NHP and LWB of piglets and are closely associated with sow reproductive performance, affecting pig production efficiency and economic profit. Hence, exploring the underlying genetic architecture of litter traits in pigs is extremely important. In this study, we explored the genetic variation of TNB, NBA, NHP and LWB in Large White pigs using GWASs and then further explored the genes related to reproduction.

Heritability of reproductive traits is low, with usually around 0.1. Previous research also reported that TNB, NBA, NHP and LWB were low heritability traits, and their heritability was approximately 0.05–0.19 in the French Large White population [1,3,40]. In our study, a linear mixed model was used to estimate genetic parameters, and the heritability of four reproductive traits ranged from 0.02 to 0.13. Furthermore, the maternal genetic effect should be considered, but there was a problem of non-convergence in the operation process when it was fitted into the model. The limited sample size may be one of the reasons according to the tutorial of blupf90. Additionally, although the genetic correlation between TNB and LWB was lower than other traits, in general, the genetic and phenotypic correlations among the four traits were highly positive and these results were consistent with the study by Wolf et al. [41].

In traditional GWAS, although we only detected five significant SNPs, it was interesting that two of them were repetitive and both located around 10.8 Mb on SSC7. In ssGWAS, the genomic regions that explained the highest genetic variance of TNB and NBA were both located at 10.72–11.06 Mb on SSC 7. Then, the region that explained the second highest genetic variance of NHP was similarly located at 10.72–11.03 Mb on SSC7 and the difference with the highest genetic variance was only 0.02%. The nearest gene around 10.8 Mb was *ENSSSCG00000044834*, but this gene has not been studied so far. Subsequently, we searched the nearby genes for this region and we finally mapped *JARID2,* which was located at 11.35–11.60 Mb on SSC 7. *JARID2* is a member of the jumonji demethylase protein family. It was reported that the protein encoded by *JARID2* interacts with the polycomb repressive complex 2 (PRC2), which plays an essential role in regulating gene expression during embryonic development [27]. Additionally, we also found four previously reported QTLs related to reproduction in this region of 0.34 Mb, including two litter size QTLs (8840, 153454) [42,43], one corpus luteum number QTL (24284) [44] and one total number born alive QTL (153455) [43]. Additionally, we detected four LD blocks in the 10.5–11.5 Mb region. We can find two SNPs (WU_10.2_7_11370371 and ASGA0031202) that are significant in traditional GWAS forming a 15K LD block and two small blocks near this block as well, so we speculate that there may be a large chain in this region that is just undetectable due to the low density of the chips we used.

Next, we totally annotated 11 candidate genes for two methods. The *GMNN* was annotated at 19.59–19.60 Mb on SSC 7 near the SNP (H3GA0020211). The *GMNN* gene encodes a protein that plays a critical role in cell cycle regulation. It was reported that deleting the *GMNN* gene in oocytes from the primordial follicle stage could impair early embryo development and implantation [28]. In TNB, NBA and LWB traits, we detected a 125.75–126.56 Mb genomic region on SSC 3, and we annotated four reproduction-related genes in this region, including *PDIA6*, *ODC1*, *KLF11* and *GRHL1*. The *PDIA6* gene is a member of the protein disulfide isomerase (PDI) family. Ye et al. showed that the *PDIA6* gene could affect oocyte quality by regulating redox homeostasis [29]. The ornithine decarboxylase 1 (*ODC1*) gene encodes the rate-limiting enzyme of the polyamine biosynthesis pathway. It was reported that *ODC1* inhibited polyamine synthesis and caused uterine quiescence, which led to entry of the blastocyst into embryo diapause [30]. It was also reported that the *KLF11* gene could adjust the expression of cytochrome (CYP) p450 enzymes to realize the genetic regulation of uterine biology [31]. The *GRHL1* gene was reported to play a critical role in placenta development [32]. Additionally, the 107.61–107.82 Mb region on SSC 7 was detected in TNB, NBA and NHP traits. In this region, we have searched the *FLRT2* gene. This gene is a member of the fibronectin leucine rich transmembrane (FLRT) family and it can regulate early embryonic vascular and neural development. It was reported that mouse embryos lacking *FLRT2* expression arrest at mid-gestation owing to cardiac insufficiency [45]. The *FLRT2* gene was also identified to be related to the NBA trait of pigs [35]. Also on SSC 7, we found the *DICER1* and *CLMN* genes in the 116.39–116.50 Mb region. The *DICER1* gene encodes a protein possessing an RNA helicase and the encoded protein functions as a ribonuclease. It has been reported that *DICER1* expression was important for early embryos in animals, including domestic livestock such as pigs and cattle [36]. In addition, knockout of *DICER1* in mouse causes morphologic abnormalities and stunted growth and lethality [38]. Mark et al. reported that the *CLMN* gene can affect the development of nervous system in early embryos [39]. There were two genes on SSC 6, *WWOX* and *UBR4*. The WWOX gene is a member of the short-chain dehydrogenases/reductases (SDR) protein family. In the study of Chen et al., the *WWOX* gene was identified as a candidate gene for calf traits in dairy cows [33]. It was reported that the *UBR4* gene played a significant role in embryonic development by affecting angiogenesis of the yolk sac [34].

## 5. Conclusions

In this study, we performed GWAS and ssGWAS analyses for TNB, NBA, NHP and LWB in French Large White pigs using a 50K SNP chip. Overall, five significant SNPs and 36 genomic regions were identified, respectively in two methods. By manually searching the literature, we identified four potential candidate genes for reproductive traits including *JARID2*, *PDIA6*, *FLRT2* and *DICER1*. Notably, in the two results, we found two overlapping SNPs (WU_10.2_7_11370371 and ASGA0031202) associated with TNB, NBA and NHP in the 10.72–11.06 Mb region on SSC 7, where we mapped the *ENSSSCG00000044834* gene. Our study screened out 11 novel genes related to reproduction and some of them have not been studied in livestock, and therefore, the function of these candidate genes needs to be further explored. The results of this study are encouraging in the search for a new major gene regulating litter size traits in pigs and provide a reference for pig genetic mechanism analysis and breeding.

## Figures and Tables

**Table 1:** Descriptive statistics of four litter traits.

Trait ^1^	Mean	SD	Min	Max	CV ^2^ (%)
TNB	14.22	3.88	3	25	27.32%
NBA	12.77	3.57	3	23	27.97%
NHP	12.00	3.37	1	23	28.10%
LWB (kg)	15.31	4.21	2.6	27.9	27.51%

^1^ TNB, total number born; NBA, number born alive; NHP, number healthy piglets; LWB, litter weight born alive. ^2^ CV, the coefficient of variation.

**Table 2:** Heritability (diagonal, bold), genetic correlation (above diagonal) and phenotypic correlation (below diagonal) for four litter traits.

Trait ^1^	TNB	NBA	NHP	LWB (kg)
TNB	**0.09 (0.04)**	0.82 (0.83)	0.78 (1.87)	0.38 (0.49)
NBA	0.85 (0.01)	**0.06 (0.04)**	0.99 (0.31)	0.70 (0.62)
NHP	0.78 (0.01)	0.93 (0.01)	**0.02 (0.03)**	0.94 (0.69)
LWB (kg)	0.68 (0.01)	0.83 (0.01)	0.85 (0.01)	**0.13 (0.05)**

^1^ TNB: total number born; NBA: number born alive; NHP, number healthy piglets; LWB: litter weight born alive.

**Table 3:** Significant SNPs for litter traits.

Trait ^1^	SNP	SSC ^2^	Position (Mbp)	*p*-Value
TNB	**WU_10.2_7_11370371**	**7**	**10.841911**	**1.77 × 10^−5^**
	H3GA0020211	7	19.658767	1.42 × 10^−5^
NBA	**WU_10.2_7_11370371**	**7**	**10.841911**	**2.09 × 10^−5^**
	**ASGA0031202**	**7**	**10.857509**	**2.33 × 10^−5^**
NHP	**ASGA0031202**	**7**	**10.857509**	**2.21 × 10^−5^**

^1^ TNB: total number born; NBA: number born alive; NHP: number healthy piglets. ^2^ SSC: the position of SNP on *Sus scrofa chromosome.*

**Table 4:** Relevant genomic regions for litter traits.

Trait ^1^	SSC ^2^	Genomic Region (Mbp)	Var (%) ^3^
TNB	3	125.756195–126.562853	3.561
	5	86.486236–86.726806	2.168
	**7**	**10.724105–11.058567**	**3.979**
	7	7:26745500–27045611	1.268
	7	107.611784–107.768080	1.354
	7	116.391085–116.501529	1.087
	8	110.219894–110.662629	2.503
	9	133.809598–134.161791	3.241
	12	54.264915–54.434349	1.080
	17	28.878576–29.033480	1.208
NBA	2	147.530240–147.685182	1.117
	3	125.756195–126.562853	3.112
	5	86.486236–86.784197	3.311
	**7**	**10.724105–11.058567**	**3.585**
	7	107.611784–107.730536	1.089
	9	133.948708–134.086750	1.076
	18	10.909022–11.384836	1.565
	18	11.574718–11.910292	2.170
NHP	2	147.400783–147.421494	1.131
	2	147.530240–147.685182	1.348
	5	86.486236–86.726806	1.621
	**7**	**10.724105–11.025466**	**2.640**
	7	71.250276–71.941528	1.105
	7	72.437167–72.997611	1.044
	7	105.990032–106.291677	2.663
	7	107.256824–107.401244	1.097
	7	107.589360–107.819775	1.895
	18	11.653824–11.778406	1.016
LWB	3	125.756195–126.562853	4.420
	5	86.178117–86.367402	1.072
	6	8.566888–9.033493	3.069
	6	77.495951–77.642338	1.071
	7	101.840965–102.117118	1.198
	11	22.269146–22.714741	1.060
	18	11.653824–11.778406	1.260
	18	40.459198–40.635413	1.011

^1^ TNB: total number born; NBA: number born alive; NHP: number healthy piglets; LWB: litter weight born alive. ^2^ SSC: the position of SNP on *Sus scrofa chromosome*. ^3^ Var (%), the highest genetic variance explained by windows among genomic regions.

**Table 5:** Genes for reproductive traits reported in previous study among candidate genes.

Method	Symbol	SSC ^1^	Position (Mbp)	Trait	Species	Publication
GWAS	*JARID2*	7	11.357961–11.602104	Early embryonic development	Bovines/mouse/pig	(Fu et al., 2017 [25]; Landeira et al., 2010 [26]; pasini et al., 2010 [27])
	*GMNN*	7	19.585999–19.599879	Embryo development and implantation	Mouse	(Ma et al., 2016 [28])
ssGWAS	*PDIA6*	3	125.751970–125.776955	Oocyte quality	pig	(Yuan et al., 2012 [29])
	*ODC1*	3	126.078418–126.132577	Oocyte physiology	Rat	(Fernandes et al., 2017 [30])
	*KLF11*	3	126.418996–126.429904	Uterine biology	Human	(Zheng et al., 2014 [31])
	*GRHL1*	3	126.464198–126.511211	Placenta development	Human	(Taracha et al., 2018 [32])
	*WWOX*	6	8.911139–9.871102	Calving	dairy cattle	(Chen et al., 2021 [33])
	*UBR4*	6	77.568415–77.707983	Yolk sac vascular development	Mice	(Tasaki et al., 2013 [34])
	*FLRT2*	7	107.812613–107.913618	Embryo development	Pig	(Bergfelder et al., 2015 [35])
	*DICER1*	7	116.361630–116.436519	Embryo development	Pig/cattle/mouse	(Kaczmarek et al., 2020 [36]; Burrola-Barraza et al., 2011 [37]; Bernstein et al., 2003 [38])
	*CLMN*	7	116.476791–116.590265	Embryo development	Human/mouse	(Marzinke et al., 2010 [39])

^1^ SSC: the position of SNP on *Sus scrofa chromosome.*

## Data Availability

The data presented in this study are available on request from the corresponding author.

## Supplementary Materials

The following supporting information can be downloaded at: https://www.mdpi.com/article/10.3390/ani12121584/s1, Table S1: windows explained >1% genetic variance for four traits, Table S2: annotation results of all genomic regions for four traits.
